# A digital embrace to blunt the curve of COVID19 pandemic

**DOI:** 10.1038/s41746-020-0279-6

**Published:** 2020-05-04

**Authors:** Lee H. Schwamm, Alistair Erskine, Adam Licurse

**Affiliations:** 1 Harvard Medical School, Boston, USA; 2 Partners Healthcare, Boston, USA; 3 Massachusetts General Hospital, Boston, USA; 4 Brigham Health, Boston, USA

**Keywords:** Health care, Health policy

## Abstract

Digital health, virtual care, telehealth, and telemedicine are all terms often used interchangeably to refer to the practice of care delivered from a distance. Because virtual care collapses the barriers of time and distance, it is ideal for providing care that is patient-centered, lower cost, more convenient and at greater productivity. All these factors make virtual care tools indispensable elements in the COVID19 response. In this perspective, we offer implementation guidance and policy insights relevant to the use of virtual care tools to meet the challenges of the COVID19 pandemic.

Digital health, virtual care, telehealth and telemedicine are all terms often used interchangeably to refer to the practice of care delivered from a distance, and for the purposes of this article we will use the umbrella term “virtual care”^1^ (Fig. 1). Because virtual care collapses the barriers of time and distance, it is ideal for providing care that is patient-centered, lower cost, more convenient and at greater productivity. There are synchronous and asynchronous modalities, and the common use cases we and others have previously implemented are captured in Fig. 1 and described in prior publications dating back over 20 years^2–6^.

**Fig. 1:**
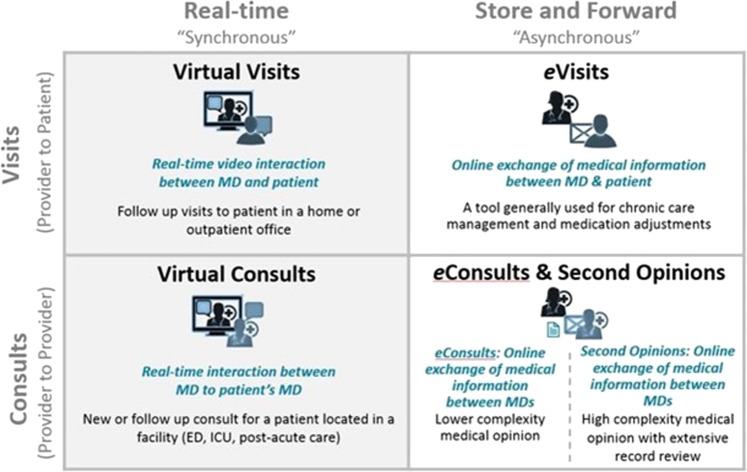
Virtual care programs at Partners Healthcare. Virtual care programs deployed at Partners Healthcare, classified by the modality of communication (synchornous vs asynchronous) and type of participants. The Virtual Visits program depicts a realtime video interaction between a provider and patient; the Virtual Consults program depicts a realtime video interaction between a referring provider and the expert teleconsultant; the eVisits program depicts a secure text-based exchange between a provider and patient; and the eConsult & Second Opinions programs depict two levels of secure text and image-based exchange between a referring provider and the expert teleconsultant, with eConsults being low complexity simple questions and Online Second Opinions being full formal consultations regarding high complexty problems and extensive records or specimens.

All these factors make virtual care a critical clinical response to the COVID19 outbreak, as has been documented in the setting of previous disasters^7,8^. We believe there is no better way to provide healthcare at scale, in a climate of social distancing for both patients and providers, than through virtual care. In order to protect patients, preserve the healthcare workforce so they can care for patients without contributing to disease spread, limit community spread to our most vulnerable patients, and prevent overcrowding across care settings.

However, multiple well-known barriers exist. Though they are rapidly evolving, insurance coverage policies have historically meant that much virtual care goes uncompensated. This lack of payment has held back development and deployment of robust virtual care offerings and capacity, such that only a small proportion of providers have been able to offer to date^9^. Fortunately, many insurers have now reduced or eliminated patient copays for virtual visits and even phone calls in some limited instances, and as a result of government decree have been forced in many settings to pay at parity with in-person rates.

Other structural barriers will need to be solved. The supply chain disruption that has not only impacted masks, gowns and other personal protective equipment (PPE) but also has also impacted the peripheral devices needed to conduct virtual care such as webcams, speakerphones, laptops, and other peripherals due to increased demand and furloughed workers in China, where many of these are manufactured. Our strategies will then need to leverage personal devices already in use by providers to connect to secure systems for communication. While telephonic assessment of many patients with mild COVID19 symptoms will be adequate for algorithm-driven, initial clinical assessment, it is not sufficient to address more complex situations. Virtual visits are necessary to fill this gap. There are consumer-facing online urgent virtual care offerings in the US and elsewhere, usually in a direct-to-consumer, fee-for-service model, but these services contribute to fragmentation of care and do not allow for documentation in the electronic medical record of the patient’s principle site of healthcare, and are not amenable to big data and natural language approaches to mapping the surges of care and load balancing across health systems. These companies of course should play a role in the COVID19 response and should consider offering their clinical services at a significant discount, and their software to providers who wish to rapidly deploy.

Medical licensure areas need to be defined more generously during this crisis. We strongly agree with action by federal and regional authorities to temporarily remove or suspend restrictions related to the need for medical licensure in the jurisdiction where the patient is located, and recommend broad extensions of malpractice insurance to cover virtual care when delivered in a clinically appropriate manner, expansion of scope of practice for all healthcare providers and staff. In addition, coverage and payment parity for virtual care should be applied to all outpatient, inpatient, and critical care encounters should be authorized for all payors for all providers. Insurance companies should deploy their increased revenue from decrease elective care utilization to increase coverage of all those providing care specific to this emergency.

At our large, academic, integrated healthcare delivery system in New England, Partners Healthcare, we have taken a virtual-first approach and are expanding our existing portfolio of highly effective virtual care solutions and retooling them to deal with many of the challenges in this pandemic. Our core principles are (1) patients first, (2) focus our provider time on delivering care, (3) keep distance whenever possible, and (4) use the simplest means possible (e.g. telephone or asynchronous messaging) when clinically appropriate. It is vital that in implementing virtual care we do not create a new social determinant of health in the form of lack of access to virtual care technology or affordability, that those with limited English proficiency be afforded the opportunity for medical interpretation, and that the visually impaired or disabled are still able to access care. For this reason, extensive use of telephonic communication may be best suited for some patients. We have identified and are implementing five priority virtual care initiatives to respond to the needs of our patients, and strongly recommend other health systems to consider these tactics as they plan their responses, now and in the future. We will offer the details of our solutions and approaches to any health system peers who request them.

1. Virtual visits: Video-enabled, provider-to-patient virtual visits will minimize in-person encounters at sites for ambulatory care for patients who would otherwise come in-person for care. This allows both providers and patients to conduct visits from their homes. This workflow is designed for patients *without* suspected COVID-19, and who are either at high risk of serious complications if infected, or who have ongoing medical conditions that will predictably deteriorate if unattended. While we have a currently active program which includes between 400 and 800 providers performing these virtual visits in a framework that is tightly integrated into our electronic health record (Epic Systems Corporation), this model is unsuitable for our rapid expansion in the coming weeks to over 11,000 providers, both fully employed by our system and private practice providers affiliated with our hospitals. For this jump in scale, we will use a standalone option outside of the immediate EHR workflow that will allow for visits to occur in a parallel channel that can be conducted on almost any device while still being secure, private, centrally managed and supported across all providers and patients. This process will also allow providers who are assigned to home quarantine due to possible exposures to continue working if they are not disabled by illness. The effort to stand up such a rapid deployment will be substantial. In our early scaled efforts these past weeks, we have seen virtual visit volume at one of our academic centers grow roughly 4000% when compared to the same period the year prior, and are seeing over 100% weekly program volume growth. We expect this level of growth to continue across the system in the near future.
2. In-room “video intercom”: This virtual solution will reduce clinical staff contact with patients who have confirmed or suspected COVID-19 illness by allowing a major reduction in the time spent in the room by providers who do not need to be providing direct care, including all staff and visitors. No patient training or device operation is needed, and the provider can connect to the patient room at any time using a standard hospital laptop or personal device. We believe this intervention will help reduce the risk of nosocomial spread as well as the rate of PPE consumption. To date, we have enabled over 1000 hospital rooms and will continue to rapidly scale as COVID cases increase and our equipment supplies allow.
3. Virtual urgent care: Video-enabled brief urgent care visits will alleviate office visits and ambulatory surge clinics when patients cannot be adequately be triaged by phone alone, to assess illness severity and need for hospitalization, provide further reassurance, and to meet other minor healthcare needs. All encounters will be documented in our electronic health record.
4. Virtual consults: This solution supports provider-to-provider expert consultation to an existing network of 30 community hospitals in New England affiliated with our health system to receive services, such as telestroke, allowing upwards of 60% of patients to remain in the community site rather than be transferred to our academic medical centers which may soon be over-capacity. We will leverage this solution to provide expert consultative care across all specialties and provider types (e.g., physicians, nurses, respiratory therapists, imaging technicians) especially critical care services that are likely going to be in high demand.
5. Automating the COVID-19 screening process: A new web-based automatic screening process leveraging a Microsoft robotic process automation tool (sometimes called a “chatbot”) and currently in use at other health systems, is being modified and implemented for use by patients and providers to help properly evaluate and classify risk of COVID-19 infection, and provide guidance on next steps in disposition. This asynchronous tool will be widely promoted to help alleviate pressure on live telephone requests to our nurse led COVID19 hotline. It will also allow for near instantaneous modification of the clinical algorithms hardwired across the system by a central update to the robotic process automation tool, which is critical in a crisis management where written and telephonic communication systems are easily overwhelmed. The week of April 13th, 9819 individuals have launched our automated Health Bot which is more than double the number of calls to our COVID-19 Nursing Triage Hotline. Of 5713 individuals who completed the Health Bot, 61% were classified as having positive symptoms and were triaged by the Bot to the Nurse Hotline, home quarantine, dedicated COVID-19 respiraory illness clinic or an emergency department.

Longer-term, as we expect a sustained response will be needed over the next year, our next wave of interventions will include provider-to-device, or remote patient monitoring at home for homebound patients currently under our care and those who are self-quarantined and at risk of deterioration, development of more robust asynchronous tools, such as structured clinical questionnaires offered via our patient portal, and enhanced remote specialty support via our large eConsult program. We firmly believe that virtual care tools, if implemented quickly and reliably, can help blunt the curve of COVID19 infection, and allow for our brick and mortar system to deliver care over more time without exceeding its capacity limits. This last point is particularly important as reported COVID-19 mortality has ranged between 0.5% and 5% in part based on healthcare delivery capacity. Two centuries ago in 1811, Drs. John Collins Warren and James Jackson called on the Boston’s gentry to help establish Massachusetts General Hospital as the state’s first public general hospital with these words, “When in distress every man becomes our neighbor, not only if he be of the household of faith, but even though his misfortunes have been induced by transgressing the rules both of reason and religion.” To meet the urgent need posed by COVID19, providers, payers, licensing boards, medicolegal carriers, and technology companies will need to break from traditional silos, re-align their incentives for the common good, and deliver on our promise to our neighbors and patients to provide patient-centered, safe, and reliable healthcare during this crisis. Several guidelines and reviews have called for broader adoption of virtual care into mainstream care delivery, but beyond telestroke systems of care large-scale uptake has been scant^10–15^. Now more than ever we need all providers to become rapidly proficient in delivering care virtually, rather than confining the practice of a small group of specialists^16^. One of the few silver linings to this health crisis is that it will transform our antiquated methods of healthcare delivery and show us new ways in which more evolved technology can truly add value to healthcare for all.
